# Evaluation of the improvement of walking ability in patients with spinal cord injury using lower limb rehabilitation robots based on data science

**DOI:** 10.1515/tnsci-2022-0320

**Published:** 2023-11-11

**Authors:** Hui Zhao, Jingyi Yang, Jie Yang, Hongying Jiang, Yecai Qin, Qian Lei

**Affiliations:** Department of Rehabilitation Medicine Center, West China Hospital, Sichuan University/West China School of Nursing, Sichuan University, Chengdu 610041, Sichuan, PR China; Key Laboratory of Rehabilitation Medicine in Sichuan Province, Chengdu 610041, Sichuan, PR China

**Keywords:** spinal cord injury, lower limb rehabilitation robot, rehabilitation training, walking ability

## Abstract

Spinal cord injury (SCI) is a serious disabling injury, and the main factors causing SCI in patients include car accidents, falls from heights, as well as heavy blows and falls. These factors can all cause spinal cord compression or even complete rupture. After SCI, problems with the movement, balance, and walking ability of the lower limbs are most common, and SCI can cause abnormalities in patient’s movement, sensation, and other aspects. Therefore, in the treatment of SCI, it is necessary to strengthen the rehabilitation training (RT) of patients based on data science to improve their motor ability and play a positive role in the recovery of their walking ability. This article used lower limb rehabilitation robot (LLRR) to improve the walking ability of SCI patients and applied them to SCI rehabilitation. The purpose is to improve the limb movement function of patients by imitating and assisting their limb movements, thereby achieving pain relief and muscle strength enhancement and promoting rehabilitation. The experimental results showed that the functional ambulation category (FAC) scale scores of Group A and Group B were 0.79 and 0.81, respectively, in the first 10 weeks of the experiment. After 10 weeks of the experiment, the FAC scores of Group A and Group B were 2.42 and 4.36, respectively. After the experiment, the FAC score of Group B was much higher than that of Group A, indicating that Group B was more effective in improving patients’ walking ability compared to Group A. This also indicated that LLRR rehabilitation training can enhance the walking ability of SCI patients.

## 1 Introduction

Data science is an interdisciplinary field that uses scientific methods, processes, algorithms, and systems to extract value from data. The application of lower limb rehabilitation robots (LLRRs) cannot be separated from data science. After spinal cord injury (SCI), it has a significant impact on patient’s life, mainly manifested as problems with walking ability. The ability to walk is not only related to patient’s ability to stand and return to family and society, but also to their confidence and quality of life. Therefore, the reconstruction of walking ability after SCI is crucial for patient’s future survival. With the rapid development of data science, intelligent rehabilitation technology has gradually taken a dominant position, and the emergence of LLRRs provides a new technological platform for the recovery of walking ability after SCI.

As the pace of human life becomes faster and faster, the pressure of work becomes greater and greater. In addition to the deterioration of living environment and the reduction of rest time, the incidence rate of SCI is becoming younger and younger. Some scholars have found that lower limb robot rehabilitation training (RT) and auxiliary equipment can improve the rehabilitation effect. Bhardwaj Siddharth found that with the increasing mobility inconvenience rate and number of SCI patients, advanced methods for providing rehabilitation and assistive services for patients have become important. He believed that although the use of robots has shown a promising method for rehabilitation and reducing the burden on nursing staff, extensive and innovative research is still needed in cognitive and physical human–machine interaction to achieve RT effectiveness and efficiency [1]. Shi proposed that the lower limb rehabilitation exoskeleton robot integrated sensing, control, and other technologies, showing the characteristics of interdisciplinary fields such as bionics, robotics, information and control science, and medicine. Since the goal of RT is to restore patient’s motor ability to a normal level, studying human gait is the foundation of research on lower limb exoskeleton rehabilitation robots [2]. Li introduced a rigid flexible coupling wearable LLRR, which was designed based on gait biomechanics and was beneficial for RT of lower limb movement disorders through gait training. This robot has the advantages of comfort, portability, and low cost and can be used for RT in medical institutions or as a daily walking assistance device for patients [3]. The purpose of Wang was to solve the problems existing in the configuration limitations, human–machine compatibility, and multimodal RT of existing LLRRs. He proposed a composite correction controller that not only ensured the tracking accuracy of LLRRs driven by cables, but also effectively improved the force accuracy of cable tension, meeting the requirements of lower limb RT [4]. Almaghout introduced a new type of exoskeleton rehabilitation robot that can be used for lower limb RT of patients after SCI. He proposed a novel design of a typical knee ankle rehabilitation robot. In order to reduce the unexpected interaction torque between the knee and ankle rehabilitation robots and patients, an admittance control algorithm was added to the controller to ensure safe RT [5]. The aforementioned scholars have proposed that the primary rehabilitation goal after SCI is to restore walking ability, which is the most urgent desire and demand of patients. Therefore, improving the lower limb walking function of SCI patients in clinical practice is the most important rehabilitation task.

SCI refers to the structural deformation of the spine and damage to certain parts of the trunk caused by external forces, resulting in abnormalities in movement, sensation, and nerves. In severe cases, SCI may result in loss of some or all of the ability to engage in daily activities, placing a significant burden on patient’s family. SCI is a serious orthopedic disease that can cause limb disability in patients. Currently, the most important method is to repair it to improve patients’ walking ability and prevent serious complications such as paralysis. However, comprehensive RT can repair incomplete SCI to a certain extent, allowing patients to gradually recover their motor and life activities and allowing some patients to take care of themselves or partially take care of themselves. In the research of modern kinematics, repeated exercises can help patients recover physically, but the efficiency is not high. The LLRR, on the other hand, focuses on a highly repetitive and precise physiological gait, providing continuous walking training to patient’s limbs, characterized by strong motor ability [6,7]. In the past decade, LLRRs have been widely used for walking ability training after SCI.

## 2 RT evaluation based on LLRRs

### 2.1 Working mode of lower limb RT robot

SCI is a highly disabling disease that can cause limb paralysis and have an impact on patient’s sensation, movement, and nerves. It can cause a series of complications such as pressure ulcers, lung infections, and urinary tract infections, seriously affecting the quality of life of patients [8]. In the process of SCI RT, there is a need to improve the walking ability of the patient, thereby enhancing their self-care ability and self-esteem. This is a key way to help patients return to society and also reduce their living burden.

At present, there are still many problems in RT in SCI, such as significant limitations on patient’s pelvic and leg mobility during walking and a lack of feedback control strategies that can adapt to external environments. These problems can only be solved with the development of science and technology. LLRR is a new bionic robot based on robotics, bionics, control theory, communication technology, and information processing technology [9,10]. The LLRR system is a gait rehabilitation system that is oriented to limb movement with joint actuation as the main purpose [11]. Rehabilitation trainers need to have a thorough understanding of robot’s devices and continuously adjust training parameters based on patient’s motor ability, so that the patient can maximize their autonomous motor ability in a precise control environment and obtain the best motor training results.

Patients with abnormal limb movements are the main users of LLRRs. The LLRR allows patients to imitate normal walking patterns and slowly restore their leg muscle strength, avoiding muscle atrophy caused by a lack of movement in the legs that have been left immobile for a long time [12]. Through computer operation, patients can imitate normal walking postures and adjust the user’s gait and foot size while walking, making their walking posture and speed closer to normal walking postures. With the intervention of modern technology such as computers and robots, lower limb rehabilitation can become more scientific and efficient. Robots have two training modes: active mode and passive mode. The combination of the two is suitable for patients at different stages of rehabilitation.

#### 2.1.1 Passive movement mode

Passive exercise is suitable for patients in the early stages of rehabilitation. Because patients in the early stages of rehabilitation have insufficient lower limb muscle strength and are unable to perform lower limb movements autonomously, it is necessary to fully rely on external forces to assist them in completing their movements [13]. The users of LLRRs are all patients with lower limb movement disorders, whose lower limbs have lost some or all of their mobility, so it is usually impossible to complete a complete RT and it is also difficult to fix the legs in a certain area [14,15]. In this context, the foot pressure sensor installed on the foot pedal senses patient’s sensory information, adjusts patient’s foot to a passive training state, and moves patient’s foot to a designated position through a gait mechanism. There are several key features worth emphasizing when using foot pressure sensors, which can provide insight into patient’s gait and movement, adjust and correct their behavior, and help improve performance and prevent injury [16].

The expected trajectory is an elliptical like shape, so its expected trajectory \[{\theta }_{d}(t)]\] is bounded. The definition of tracking error \[\mathop{e}\limits^{\ast }]\] can be expressed as:

(1) \[\left\{\begin{array}{c}e(t)=\theta (t)-{\theta }_{d}(t)\\ \mathop{e}\limits^{\ast }(t)=\mathop{\theta }\limits^{\ast }(t)-{\mathop{\theta }\limits^{\ast }}_{d}(t),\end{array}\right.]\]

where \[\theta (t)]\] represents the actual trajectory of the robot in the sagittal plane, and \[\mathop{\theta }\limits^{\ast }(t)]\] represents the expected trajectory of the robot in the sagittal plane. By introducing *v*, a controller can be obtained in the following form:

(2) \[\tau =D(\theta )v+h(\theta ,\mathop{\theta }\limits^{\ast })+G.]\]

It is substituted into the formula to obtain:

(3) \[D(\mathop{\theta }\limits^{\ast })v=0.]\]

If *D* is reversible, it can be obtained from the formula:

(4) \[\mathop{\theta }\limits^{\ast }=v.]\]

Based on the positive qualitative analysis of \[{K}_{v}]\] and \[{K}_{p}]\] , \[(\theta ,\mathop{\theta }\limits^{\ast })]\] is obtained as the equilibrium point, and the passive training mode of the LLRR can be obtained. The expression is as follows:

(5) \[\tau =D(\theta )(\mathop{{\theta }_{d}}\limits^{\ast }-{K}_{v}\mathop{e}\limits^{\ast }-{K}_{p}e)+h(\theta ,\mathop{\theta }\limits^{\ast })+G(\theta ).]\]

In previous RT, patients needed to achieve recovery through limb stretching and bending under the guidance of caregivers, but because each therapist’s movements and strength were different, it also had a significant impact on patient’s movements. In the long run, patients’ recovery would not only fail to improve, but there would also be secondary injuries, which means that patient’s body would have to bear more pressure and would also bring more financial burden to patient’s family [17]. In terms of structure, the LLRR connects the output end to the human feet, and the human body stands on the pedal using an electric motor to drive the crank, allowing patient’s lower limbs to undergo cyclic RT [18]. At this point, it is necessary for the patient to have some control over the pelvis, and even when the body cannot support it, suspension can be used to balance patient’s body weight, thereby reducing patient’s burden. During RT, patients can choose different gears based on their physical condition to practice at different speeds and trajectories. During the entire rehabilitation process, as long as the training mode is set, the gait mechanism of the LLRR can be directly controlled by a computer for movement, making patient’s RT process simpler and the rehabilitation effect more scientific and effective.

#### 2.1.2 Active exercise mode

The active training of LLRRs is mainly aimed at patients with certain lower limb mobility. This group of people has lower limb mobility compared to normal individuals, and their reaction ability is correspondingly delayed. Their body coordination and balance abilities are also poor [19,20]. Traditional RT methods are carried out with the assistance of accompanying personnel. However, without proper assistance, it is likely to lead to secondary injuries. The use of scientifically designed LLRRs allows patients to actively engage in RT according to their own exercise preferences, greatly improving the effectiveness of rehabilitation. At the beginning, the patient is guided by a walking mechanical device to simulate walking in a circular manner. When the patient reaches a certain level, their muscle strength would be restored and they can perform some active movements. The patient can then conduct scientific and effective active training based on their actual situation.

The LLRR is also equipped with an incremental photoelectric encoder and controller in the transmission components. The incremental photoelectric encoder can detect and track the state of patient’s two feet moving with the pedal in real time, and the controller would track and control the patient in real time according to their movement intensity. Therefore, in this active RT mode, the walking path of the patient is monitored and planned by inputting information such as impedance and plantar force. Based on the detected crank angle, the parameters of the real-time motion state of the patient are obtained, and control variable \[e(t)]\] is obtained. The mechanical device that enables human walking through position control can track the feet of the patient in real time.

According to the process of the RT mode mentioned earlier, it is concluded that in the active RT mode, the output torque \[\varpi ]\] of the motor is controlled through position deviation, with the aim of driving the pedal to move in a specific trajectory. The relationship expression between \[e(t)]\] and \[\varpi ]\] is as follows:

(6) \[\varpi ={K}_{p}\cdot e(t)+{K}_{v}\cdot \underset{0}{\overset{t}{\int }}e(t)\text{d}t.]\]

During training, the patients should be allowed to exercise in a comfortable environment. While losing weight, it is important to maintain balance in order to better simulate the walking posture of a normal person. When using external forces for RT, it is important to pay attention to safety and comfort and try not to make too large movements to avoid muscle strain and joint damage. When the muscle strength of the patient is insufficient to bear their own weight, a weight loss system needs to be used to assist the patient in exercise. The weight loss plan of the LLRR uses a suspension weight loss mode similar to a single rope.

When a patient experiences excessive upper body movements during training, resulting in a deviation of the rope from the vertical direction, the relationship between the tension of the rope and the reduction of gravity is as follows:

(7) \[F=\frac{{F}_{v}}{\cos \hspace{.25em}x},]\]

where \[x]\] represents the angle between the center of the circle and the line connecting the contact point between the rope and the person and the vertical direction. The length of the rope is much larger than the outer diameter of the pulley, and the size of the latter can be ignored, which can have a negligible impact on the calculation results. The angle between the line connecting center \[{b}_{A1}]\] and the contact point \[{b}_{A2}]\] between the rope and the person and the vertical direction is as follows:

(8) \[x=\arccos \left(\frac{| {b}_{A1}-{b}_{A2}| }{\sqrt{{({a}_{A1}-{a}_{A2})}^{2}}+\sqrt{{({b}_{A1}-{b}_{A2})}^{2}}+\sqrt{{({z}_{A1}-{z}_{A2})}^{2}}}\right).]\]

When the rope is under tension, its horizontal component \[{F}_{h}]\] is expressed as:

(9) \[{F}_{h}=f\hspace{.25em}\sin \hspace{.25em}x.]\]

To enable patients to comfortably perform weight loss training on LLRRs, it is necessary to minimize the horizontal component of the rope on the patient and avoid the adverse effects of the horizontal component on patient’s gait training. While designing, the angle should be adjusted to the minimum, such as installing the pulley at a higher position.

### 2.2 Application of LLRRs in SCI

Rehabilitation robots are an important research topic to improve patients’ daily mobility, enhance their self-care ability, and enhance their quality of life. It can be divided into two categories: one is RT type, and the other is used for auxiliary rehabilitation. The role of RT robots can help patients complete various active and passive RT, thereby reducing the workload of rehabilitation trainers. At the same time, it can also solve the problem of manual assistance training not achieving long-term activity of all muscles and joints throughout the body, such as walking training, hand and upper limb exercise training, spinal exercise training, and neck exercise training.

#### 2.2.1 Application in patients with complete SCI

There have been many reports on the RT of SCI regarding its walking function, and LLRRs have been widely used in SCI RT. For patients with incomplete and completeness SCI, LLRR can effectively improve walking ability and reduce expenditure. It can be foreseen that in the near future, there would be greater development space for LLRRs.

At present, the research on LLRRs is relatively mature. SCI patients can stand and walk under LLRRs, but their connection with daily life still needs further exploration. At present, other countries have attempted to use soft robots for SCI patients, which can improve their range of motion and comfort. However, the selection of material rigidity remains a challenge.

#### 2.2.2 Application in patients with incomplete SCI

For the incomplete SCI training, it can be divided into two types: one is the basic training focusing on improving the muscle strength and joint function of the limbs, and the other is the overall training of the walking function, which aims to improve the balance and coordination ability of the patients during walking.

Basic training: currently, there are various types of robots used for basic lower limb motion training. Although most robots can perform assisted, passive, and resistant exercise training, their disadvantage lies in the difficulty in reproducing the motion training of rehabilitation physicians, which brings more humanized guidance to patients. They are mainly used for auxiliary, resistance, and passive motion modes of the knee and ankle joints.

Overall training of walking function: due to the low lower limb motor ability of most SCI patients, they are unable to bear the weight of normal individuals. Therefore, weight loss measures must be taken to achieve weight loss and achieve better rehabilitation effects, which can provide continuous and standardized walking exercise for patients. The walking speed and weight loss of the LLRR are the main parameters to be adjusted during this process. During the walking process, the magnitude of weight loss should be stable, without any special requirements for walking speed. The main goal of walking training for SCI patients is to restore their normal gait, so gait planning is crucial. Two different gait RT methods are usually used for the characteristics of limb movements after SCI. One is passive RT, and the other is cooperative RT.

After the recovery of lower limb muscle strength, patients would experience discomfort with walking methods, and coordinated gait training is required to enable patients to achieve correct walking under the guidance of robots. Proactivity and interaction are key links in walking training. As patients walk more freely, their muscle activity during the walking process would change, forming a continuous feedback of information, thereby achieving the reconstruction of brain and spinal neural networks and achieving good therapeutic effects.

## 3 Evaluation of the improvement effect of LLRRs on walking ability of SCI patients

### 3.1 Experimental data and indicators

#### 3.1.1 Research objects and methods

Forty SCI patients admitted to the Rehabilitation Department of a certain People’s Hospital from January 2021 to December 2021 were selected and divided into a conventional RT group (Group A) and a LLRR RT group (Group B), each with 20 patients. The basic information of the patient is shown in Table 1:

**Table 1:** Basic information of patients

Situation	Type	Group A	Group B
Gender	Male	12	11
	Female	8	9
Age (year)	15–25	7	6
	Above 25	13	14
Course of the disease (month)	1–6	15	16
	Above 6	5	4

As shown in Table 1, there were more males than females in the two groups, with more patients over the age of 25 and more patients with a disease course of 1–6 months. The difference between the two groups was very small.

Routine RT group (Group A): the number of exercises per week was maintained at 5. It lasted for 30 min per session and was practiced continuously for 10 weeks. Patients between the two groups cannot be interchanged, and blood pressure should be measured before each RT. The purpose of measuring blood pressure is to ensure that patient’s blood pressure is within a safe range and then perform corresponding exercises. At the same time, it is also necessary to do well in bed and out of bed training.

LLRR RT group (Group B): Group B provided LLRR RT on the basis of routine RT and needed to set weight reduction, walking speed, and guidance force during training. The training was maintained at a normal rate of 5 times per week and 30 min per session. Two groups of patients underwent 10 weeks of RT.


**Informed consent:** Informed consent has been obtained from all individuals included in this study.
**Ethical approval:** The research related to human use has been complied with all the relevant national regulations, institutional policies, and in accordance with the tenets of the Helsinki Declaration, and has been approved by the authors’ institutional review board or equivalent committee.

#### 3.1.2 Evaluation indicators

The functional walking category scale (FAC) was used to evaluate the walking ability of two groups of patients. The American Spinal Injury Association (ASIA) was used to evaluate the severity of SCI in patients. The Berg Balance Scale (BBS) was used to evaluate the walking balance ability of patients, and the lower limb Fugl–Meyer assessment (FMA) was used to evaluate the lower limb motor function of patients. FMA was more sensitive and accurate in describing the level and degree of injury and was applicable to all patients.

### 3.2 Comparison of different RT methods

#### 3.2.1 Walking ability rating

Most SCI patients suffer from walking impairments, so how to restore their walking ability is a major goal of RT. Due to differences in the etiology, location of injury, and severity of each SCI patient, decreased muscle strength, high lower limb muscle tension, pelvic forward tilt, pelvic retraction, foot drop, pelvic instability, and knee overextension can all cause abnormal gait. To improve patient’s walking ability, it is necessary to carry out muscle strength training, standing training, rotation training, balance training, and walking training for the patient.

**Figure 1:**
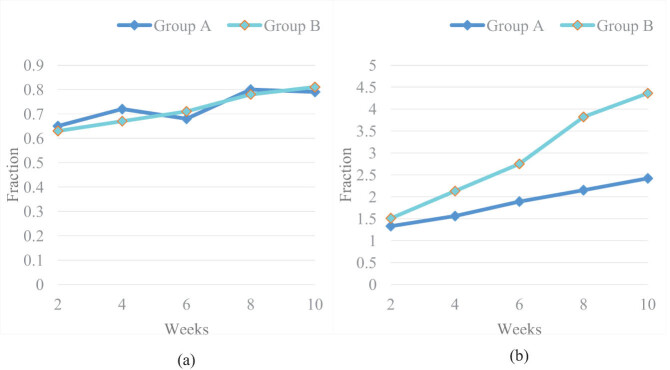
FAC scores of the two groups before and after the experiment: (a) FAC scores of the two groups before the experiment and (b) FAC scores of the two groups after the experiment.

The principle of RT is to enhance patient’s muscle strength, reduce muscle tension, and enhance patient’s balance and coordination, so that the patient can control their gait well. In clinical practice, FAC was the most commonly used scale to evaluate patients’ RT walking ability, which was divided into 0–5 levels. In order to make the experiment more clear, this article assumed a score of 0–5 for the 0–5 level. The higher the score, the stronger the walking ability. Therefore, FAC scores were given to the two groups of patients. The FAC scores of two groups of patients before and after RT were collected, as shown in Figure 1:

It can be seen from the data in Figure 1(a) that the FAC scores of Group A and Group B were both very low in the first 2 weeks of the experiment, with scores of 0.65 and 0.63, respectively. In the first 10 weeks of the experiment, the FAC scores of Group A and Group B were also very low, with scores of 0.79 and 0.81, respectively. Before the experiment, the walking function of Group A and Group B was very poor.

From Figure 1(b), it was found that after the experiment, the FAC scores of Group A and Group B were 1.33 and 1.51, respectively. Compared to before the experiment, the FAC scores of both groups improved, indicating an improvement in walking function in both groups. At 10 weeks after the experiment, the FAC scores of Group A and Group B were 2.42 and 4.36, respectively. At this time, the FAC scores of Group B were much higher than those of Group A.

The results showed that the FAC scores of both groups after training have significantly improved compared to before, and the scores of Group B were significantly better than those of Group A. Although traditional conventional rehabilitation methods can improve the walking function of SCI patients, their efficacy was far inferior to that of LLRRs. LLRRs can improve patients’ sense of space, attention, execution, and memory and can achieve twice the result with half the effort, greatly accelerating the recovery of patients’ walking ability.

The training of LLRRs does not require high physical fitness. As long as the situation is stable, it can be carried out. Compared with previous specialized training to improve the patient muscle strength and balance, LLRR training can allow patients to continuously repeat the entire composite movement of the walking cycle, thereby achieving the goal of improving walking ability. Moreover, LLRR training belongs to quantitative, timed, repetitive, and progressive training, which can ensure that the patients can maintain consistent and sustained training.

#### 3.2.2 ASIA score comparison

The ASIA score can better reflect the SCI status of the patients, as well as their motor, sensory function, and rehabilitation status. The total score of the motor score was 0–70 points, and the total score of the sensory (tactile sensation and pain perception) score was 29–87 points. A higher tactile score indicates an improvement in the tactile function of the patients, while a lower pain score indicates the lower pain. The ASIA scores of the two groups before the experiment are shown in Table 2.

**Table 2:** ASIA scores of the two groups before the experiment

Types	Group A	Group B
Exercise	43.26	42.98
Tactile sensation	65.41	65.53
Pain perception	44.72	44.69

As shown in Table 2, the ASIA scores for exercise, tactile sensation, and pain perception in Group A before the experiment were 43.26 points, 65.41 points, and 44.72 points, respectively. The ASIA scores for exercise, tactile sensation, and pain perception in Group B before the experiment were 42.98, 65.53, and 44.69, respectively. Before the experiment, there was no significant difference in ASIA scores for exercise, tactile sensation, and pain perception between Group A and Group B, and both were not high.

The ASIA scores of the two groups after the experiment are shown in Table 3.

**Table 3:** ASIA scores of the two groups after the experiment

Types	Group A	Group B
Exercise	47.37	65.09
Tactile sensation	71.05	83.32
Pain perception	37.22	30.24

As shown in Table 3, the ASIA scores for exercise, tactile sensation, and pain perception in Group A after the experiment were 47.37 points, 71.05 points, and 37.22 points, respectively. After the experiment, the ASIA scores for exercise, tactile sensation, and pain perception in Group B were 65.09, 83.32, and 30.24, respectively.

Table 3 shows that after RT, the exercise, pain perception, and tactile sensation of Group A and Group B have improved compared to before RT, while the improvement of exercise, pain perception, and tactile sensation in Group B was significantly better than that in Group A.

#### 3.2.3 Balance ability comparison

To maintain the correct posture, the body must have good balance ability in order to ensure the normal life of patients. Therefore, this is also an important indicator for evaluating the physical activity ability of SCI patients, which can effectively restore body balance and is very important for patients to return to life. Balance ability is controlled by the central nervous system, which coordinates various movements to achieve changes in body posture, and makes timely adjustments when the center of gravity deviates, enabling the body to maintain multiple postures and achieve corresponding actions. In the early stage of SCI, patients’ normal walking ability is almost completely lost, and they are unable to control and maintain their body posture. In order to enable patients to walk normally, it is necessary to provide balance and coordination training to the patients. The application of BBS in the evaluation of balance ability is becoming increasingly widespread, and its reliability and validity are also increasing. BBS has a total of 14 projects, with a total score of 56 points. The higher the score, the stronger the balance ability.

**Figure 2:**
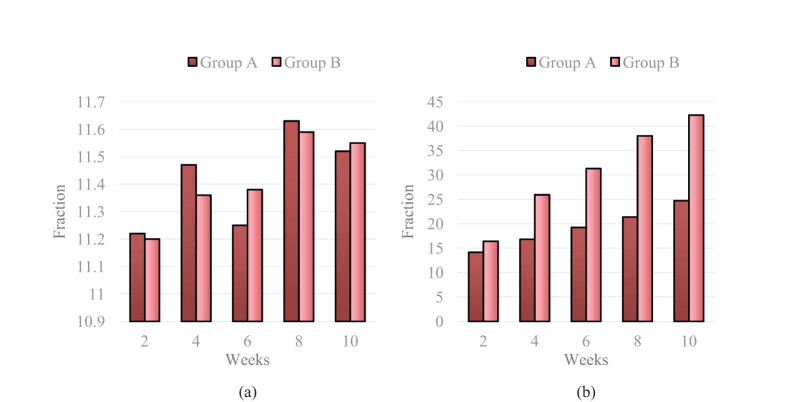
BBS scores of two groups before and after the experiment: (a) BBS scores of the two groups before the experiment and (b) BBS scores of the two groups after the experiment.

In the experiment, two groups of patients were compared, and the BBS scores of patients in both groups before and after RT are shown in Figure 2.

Through the analysis of the BBS scores of Group A and Group B before the experiment in Figure 2(a), it can be found that the BBS scores of Group A and Group B were 11.22 and 11.20 points, respectively, in the first 2 weeks of the experiment, and the BBS scores of Group A and Group B were 11.52 and 11.55 points, respectively, in the first 10 weeks of the experiment. The difference in BBS scores between the two groups before the experiment was not significant and both groups were very low, indicating that the balance ability of the two groups was not high at this time.

After analyzing the data in Figure 2(b), it was found that after the experiment, the BBS scores of Group A and Group B were 14.16 and 16.41, respectively. At this time, the BBS scores of both groups were improved compared to before the experiment, but the improvement was not significant. After 10 weeks of the experiment, the BBS scores of Group A and Group B were 24.72 and 42.27, respectively. Compared with the two groups before the experiment, the improvement in BBS scores was significant, and the BBS score of Group B was much higher than that of Group A.

By comparing and analyzing the BBS scores of two groups of patients before and after RT, it was proven that the RT plan of Group B had a significant effect on improving the balance function of SCI patients.

#### 3.2.4 Comparison of lower limb motor function

**Figure 3:**
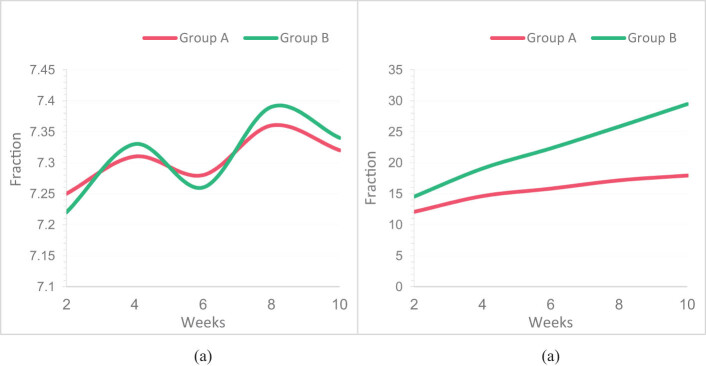
FMA scores of two groups before and after the experiment: (a) FMA scores of the two groups before the experiment and (b) FMA scores of the two groups after the experiment.

FMA, as an important method for evaluating lower limb motor function, has a total of 17 items. Each item has a maximum score of 2 points, totaling 34 points. The higher the score, the better the motor function of the lower limbs. This article statistically analyzed the FMA scores of two groups of patients before and after RT. The FMA scores of the two groups before and after the experiment are shown in Figure 3.

It was observed in Figure 3(a) that the FMA scores of Group A and Group B were 7.25 and 7.22, respectively, in the first 2 weeks of the experiment. At this time, the FMA score of Group B was slightly lower than that of Group A. The FMA scores of Group A and Group B were 7.32 and 7.34, respectively, in the first 10 weeks of the experiment. At this time, the FMA scores of Group A and Group B were not significantly different, indicating that the FMA scores of the two groups were similar before the experiment.

According to Figure 3(b), after the experiment, the FMA scores of Group A and Group B were 12.07 and 14.52, respectively, with higher scores in both groups than before the experiment. After 10 weeks of the experiment, the FMA scores of Group A and Group B were 17.93 and 29.46, respectively. The FMA scores of Group B were significantly increased, and the gap between Group A and Group B was widened.

Through the FMA score in Figure 3, it is found that LLRR training in Group B has a significant effect on improving lower limb motor function in SCI patients. Group B is more effective than Group A in improving the lower limb motor function of patients, which is likely due to the LLRR being able to control patient’s limb movements and having a unique feedback and control system that allows patients to imitate normal walking patterns.

## 4 Conclusions

SCI is a relatively common trauma. Most patients have left a certain degree of walking problems. Especially for patients with completeness SCI, patients cannot stand up or walk. How to restore patients’ walking ability is still an urgent problem to be solved. Most of the existing LLRRs can help SCI patients regain walking ability. This article aimed to compare conventional training and LLRR training for SCI patients, in order to find a more ideal RT method and apply it to clinical practice. The experimental results indicated that the effectiveness of LLRR training was significantly higher than that of conventional training in terms of lower limb motor function, balance ability, walking ability, etc. Of course, in order to successfully use LLRRs, it is necessary to fully consider the special circumstances of patients and conduct standardized and systematic comprehensive RT. Rehabilitation robots are a new type of rehabilitation tool that has obvious advantages compared to traditional rehabilitation. They can provide long-term and stable training for patients, and objectively evaluate the results of RT, thereby reducing the workload of rehabilitation trainers and improving work efficiency. Therefore, they should be popularized to more patients.
